# Treatment patterns and clinical outcomes in patients with advanced non-small cell lung cancer initiating first-line treatment in the US community oncology setting: a real-world retrospective observational study

**DOI:** 10.1007/s00432-020-03414-4

**Published:** 2020-12-02

**Authors:** Eric Nadler, Bhakti Arondekar, Kathleen Marie Aguilar, Jie Zhou, Jane Chang, Xinke Zhang, Vivek Pawar

**Affiliations:** 1 Texas Oncology-Baylor Charles A. Sammons Cancer Center, Dallas, TX USA; 2 McKesson Life Sciences, 10100101 Woodloch Forest Dr, The Woodlands, TX USA; 3 Pfizer Inc, New York, NY USA; 4 EMD Serono Research & Development Institute, Inc., Billerica, MA, USA; an affiliate of Merck KGaA, Darmstadt, Germany, Billerica, MA USA; 5 Merck KGaA, Darmstadt, Germany

**Keywords:** Electronic health record, Immuno-oncology, Overall survival, Time to treatment discontinuation, Community oncology

## Abstract

**Purpose:**

Treatments for advanced non-small cell lung cancer (NSCLC) have evolved to include targeted and immuno-oncology therapies, which have demonstrated clinical benefits in clinical trials. However, few real-world studies have evaluated these treatments in the first-line setting.

**Methods:**

Adult patients with advanced NSCLC who initiated first-line treatment with chemotherapy, targeted therapies (TT), or immuno-oncology–based regimens in the US Oncology Network (USON) between March 1, 2015, and August 1, 2018, were included and followed up through February 1, 2019. Data were sourced from structured fields of USON electronic health records. Patient and treatment characteristics were assessed descriptively, with Kaplan-Meier methods used to evaluate time-to-event outcomes, including time to treatment discontinuation (TTD) and overall survival (OS). Adjusted Cox regression analyses and inverse probability of treatment weighting (IPTW) were performed to control for covariates that may have affected treatment selection and outcomes.

**Results:**

Of 7746 patients, 75.6% received first-line systemic chemotherapy, 11.7% received immuno-oncology monotherapies, 8.5% received TT, and 4.2% received immuno-oncology combination regimens. Patients who received immuno-oncology monotherapies had the longest median TTD (3.5 months; 95% confidence interval [CI], 2.8–4.2) and OS (19.9 months; 95% CI, 16.6–24.1). On the basis of multivariable Cox regression and IPTW, immuno-oncology monotherapy was associated with reduced risk of death and treatment discontinuation relative to other treatments.

**Conclusion:**

These results suggest that real-world outcomes in this community oncology setting improved with the introduction of immuno-oncology therapies. However, clinical benefits are limited in certain subgroups and tend to be reduced compared with clinical trial observations.

**Electronic supplementary material:**

The online version of this article (10.1007/s00432-020-03414-4) contains supplementary material, which is available to authorized users.

## Introduction

The treatment landscape for advanced, unresectable, and/or metastatic non-small cell lung cancer (NSCLC) is evolving. Although doublet and triplet systemic chemotherapies were the mainstay treatment of advanced NSCLC for decades, limited improvements in survival rates and high occurrence of toxicity have been noted (Azim et al. 2009, 2016; Bittoni et al. 2018; Kaniski et al. 2017; Xia et al. 2017). An analysis of the Surveillance, Epidemiology, and End Results Program (SEER) database indicated that 5-year survival rates for patients diagnosed with metastatic NSCLC increased from 2.0% (95% confidence interval [CI], 1.80–2.20%) to only 4.2% (95% CI, 4.10–4.40%) between 1988 and 2008 (Xia et al. 2017). Another evaluation of the SEER database showed that the median overall survival (OS) in patients with stage IIIB/IV NSCLC was 4 months in 1996 and 5 months in 2005 and 2010 (Kaniski et al. 2017). A systematic review of 8 clinical trials published between 2000 and 2008 revealed that median OS associated with doublet or triplet therapy for advanced NSCLC was 37 and 42 weeks, respectively (Azim et al. 2016). Additionally, both regimens are associated with a high occurrence of adverse events (Azim et al. 2009, 2016; Bittoni et al. 2018).

Greater insight into the molecular mechanisms of NSCLC has resulted in the identification of prognostic biomarkers and the development of targeted therapies associated with improved clinical outcomes and safety profiles compared with systemic chemotherapy (NCCN Guidelines for Non-Small Cell Lung Cancer 6.2020). Several targeted therapies have demonstrated clinical benefits for patients with specific biomarkers, and current National Comprehensive Cancer Network (NCCN) guidelines (version 6.2020) recommend these regimens as first-line (1L) treatment for patients with sensitizing epidermal growth factor receptor (*EGFR*), anaplastic lymphoma kinase (*ALK*), ROS proto-oncogene 1 receptor tyrosine kinase (*ROS1*), proto-oncogene B-Raf (*BRAF*) V600E, neurotrophic tyrosine kinase (*NTRK*) gene fusion, or programmed death-ligand 1 (PD-L1) biomarkers.

Until recently, treatment options for patients without these biomarkers were limited to traditional systemic chemotherapies. On the basis of specific biomarkers, NCCN guidelines (version 6.2020) provide specific treatment recommendations for targeted therapies. For example, recommended 1L therapies for patients with sensitizing *EGFR* mutations include osimertinib (preferred, category 1), erlotinib (alone or with ramucirumab or bevacizumab), afatinib, gefitinib, and dacomitinib.(NCCN Guidelines for Non-Small Cell Lung Cancer 6.2020) In addition to conventional targeted therapies, immuno-oncology (IO) agents are now recommended for patients with PD-L1 expression and favorable performance status (PS). For patients with PD-L1–positive NSCLC (≥ 50% expression) without other prognostic biomarkers, pembrolizumab and atezolizumab monotherapies, along with platinum-based IO combination therapy, are preferred 1L treatments in the current NCCN guidelines (version 6.2020). IO combination therapies that include pembrolizumab, atezolizumab, or nivolumab are also recommended for patients with PD-L1 expressions of less than 50% who lack other prognostic biomarkers.

IO agents, either alone or in combination with other therapies, have demonstrated promising results in clinical trials, even in patients who lack the PD-L1 mutation (Table 1). KEYNOTE-024 and  – 042 showed superior efficacy of pembrolizumab monotherapy compared with chemotherapy, and KEYNOTE-189 and  – 407 showed superior efficacy of pembrolizumab and chemotherapy compared with placebo and chemotherapy (Gadgeel et al. 2020; Gandhi et al. 2018; Mok et al. 2019a, b; Paz-Ares et al. 2018; Reck et al. 2016, 2019a, b). IMpower131 and 132 showed superior efficacy of atezolizumab and carboplatin/paclitaxel over carboplatin/paclitaxel alone, and IMpower150 showed superior efficacy of atezolizumab, bevacizumab, and carboplatin/paclitaxel over bevacizumab and carboplatin/paclitaxel alone (Jotte et al. 2018, 2019; Papadimitrakopoulou et al. 2018; Socinski et al. 2018a, b). CheckMate 227 demonstrated superior efficacy of nivolumab plus ipilimumab over platinum doublet chemotherapy (Hellmann et al. 2018, 2019).

**Table 1:** Clinical outcomes of key clinical trials of systemic chemotherapies and IO-based regimens

	Regimens	Age, median, years	Stage	ECOG PS 0/1	PD-L1 threshold for inclusion	Follow-up, median, months	OS, median (95% CI), months			PFS/TTD, median (95% CI), months		
							Chemo	IO mono	IO combo	Chemo	IO mono	IO combo
This study	Chemo (*n* = 5859) IO mono (*n* = 907) IO combo (*n* = 324)	68.0 71.0 68.0	IV or unresectable	64.4% 62.3% 64.5%	None	7.3 6.7 5.9	14.9 (14.1– 15.7)	19.9 (16.6–24.1)	14.0 (10.4– 20.9)	TTD: 2.0 (1.9–2.1)	TTD: 3.5 (2.8–4.2)	TTD: 3.0 (2.5–3.5)
KEYNOTE-024 (Reck et al. 2016, 2019a,b)	Pembro monotherapy (*n* = 154) Chemo (*n* = 151)	64.5 66.0	IV	99.4% 100%	≥ 50%	11.2–44.4	14.2 (9.8– 18.3)	26.3 (18.3– 40.4)	–	PFS: 6.0 (4.2–6.2)	PFS: 10.3 (6.7–NR)	–
KEYNOTE-042 (Mok et al. 2019a, b)	Pembro monotherapy (*n* = 637) Chemo (*n* = 637)	63.0	IV or unresectable	100%	≥ 1%	14	12.1 (11.3– 13.3)	16.4 (14.0– 19.7)	–	PFS: 6.6 (6.3–7.3)	PFS: 5.4 (4.3–6.2)	–
IMpower110 (Spigel et al. 2019)	Atezo monotherapy (*n* = 277) Chemo (*n* = 277)	–	IV	100%	≥ 1%	15.7	17.5	14.1	–	–	–	–
KEYNOTE-189 (nonsquamous population)(Gadgeel et al. 2020)	Pembro + chemo (*n* = 410) Pembro + chemo (*n* = 206)	65.0 63.5	IV	99.8% 100%	None	23.1	10.7 (8.7–13.6)	–	22.0 (19.5–25.2)	PFS: 9.0 (8.1–9.9)	–	PFS: 4.9 (4.7–5.5)
KEYNOTE-407 (squamous population)(Paz-Ares et al. 2018)	Pembro + chemo (*n* = 278) Pembro + chemo (*n* = 281)	65.0 65.0	IV	100%	None	7.8	11.3 (9.5–14.8)	–	15.9 (13.2–NR)	PFS: 4.8 (4.3–5.7)	–	PFS: 6.4 (6.2–8.3)
IMpower130 (West et al. 2019)	Atezo + chemo (*n* = 451) Chemo (*n* = 228)	64.0 65.0	IV	100%	None	18.5 13.9	13.9 (12.0–18.7)	–	18.6 (16.0–21.2)	PFS: 5.5 (4.4–5.9)	–	PFS: 7.0 (6.2–7.3)
IMpower131 (squamous population)( Jotte et al. 2018, 2019)	Atezo + chemo (*n* = 343) Chemo (*n* = 340)	65.0	IV	100%	None	25.5	13.5 (12.2–15.1)	–	14.2 (12.3–16.8)	PFS: 5.6 (5.5–5.7)	–	PFS: 6.3 (5.7–7.1)
IMpower132 (Papadimitrakopoulou et al. 2018)	Atezo + chemo (*n* = 292) Chemo (*n* = 286)	64.0 63.0	IV	100%	None	14.8	13.6 (11.4–15.5)	–	18.1 (13.0–NR)	PFS: 5.2 (4.3–5.6)	–	PFS: 7.6 (6.6–8.5)
IMpower150 (Socinski et al. 2018a, b)	Atezo + Bev + Carbo/pac ( *n* = 356) Bev + Carbo/pac(*n* = 336)	63.0 63.0	IV or unresectable	100%	None	15.4 15.5	14.7 (13.3–16.9)	–	19.2 (17.0–23.8)	PFS: 6.8 (6.0–7.1)	–	PFS: 8.3 (7.7–9.8)
CheckMate 227 (Hellmann et al. 2018, 2019)	Nivo + Ipi (*n* = 583) Platinum doublet chemo (*n* = 583)	64.0 64.0	IV or unresectable	100%	≥ 1%	29.3	13.9 (12.2–15.1)	–	17.1 (15.2-19.9)	PFS: 5.5 (4.6–5.6)	–	PFS: 4.9 (4.1–5.6)

*Atezo* atezolizumab, *Bev* bevacizumab, *Carbo/pac* carboplatin/paclitaxel, *chemo* chemotherapy, *CI* confidence interval, *combo* combination regimen, *ECOG PS* eastern cooperative oncology group performance status, *IO* immuno-oncology, *Ipi* ipilimumab, *mono* monotherapy, *Nivo* nivolumab, *NR* not reached, *OS* overall survival, *PD-L1* programmed death ligand-1, *Pembro* pembrolizumab, *PFS* progression-free survival, *TTD* time to treatment discontinuation

On the basis of trial results, NCCN guidelines (version 6.2020) recommend IO regimens as 1L treatment for patients with advanced NSCLC without other prognostic biomarkers, according to their histology results and PS. (NCCN Guidelines for Non-Small Cell Lung Cancer 6.2020). For patients with Eastern Cooperative Oncology Group (ECOG) PS of 0/1 and adenocarcinoma, large cells, or another histology not otherwise specified, pembrolizumab alone or in combination with pemetrexed and either carboplatin or cisplatin, as well as atezolizumab monotherapy, are preferred regimens. For patients with squamous cell carcinoma and an ECOG PS of 0/1, pembrolizumab alone or in combination with carboplatin and paclitaxel, as well as atezolizumab monotherapy, are preferred regimens.

Since the US Food and Drug Administration’s approval of IO therapies for advanced NSCLC, the use of these treatments has increased in the US community oncology setting (Khozin et al. 2019b). However, few studies have examined the real-world treatment patterns and clinical outcomes of these therapies. These studies have mostly included patients with previously treated advanced NSCLC, lacked comparisons between treatments, and/or limited the patient population to those with PD-L1 expression (Khozin et al. 2018; Molife et al. 2019; Nadler et al. 2018; Schwartzberg et al. 2019; Velcheti et al. 2019; Weis et al. 2019). Further research on IO use in patients with advanced NSCLC treated in the community oncology setting is needed, as there may be differences in real-world clinical outcomes compared with clinical trial outcomes due to underlying variation in patient populations and methodology (Khozin et al. 2019a).

The aim of this study was to provide real-world insight into contemporary treatment patterns and outcomes in patients with advanced NSCLC who initiated 1L treatment within a large network of US community oncology practices. This insight will help providers and health benefits administrators navigate complex treatment pathways to determine appropriate regimens based on patient and disease characteristics. In addition to examining how IO therapies may have changed the treatment landscape, we compared clinical outcomes across treatment types.

## Patients and methods

### Study design and data sources

This was a retrospective, observational, descriptive study of adult patients with advanced NSCLC who initiated 1L treatment with systemic chemotherapy, targeted therapies, or IO-based regimens between March 1, 2015, and August 1, 2018, within the US Oncology Network (USON). Initiation of 1L treatment was considered the index event, and eligible patients were followed up through February 1, 2019. Patients younger than 18 years at diagnosis, those with fewer than 2 visits after index, clinical trial participants, and those with another documented primary cancer were excluded. Additionally, patients with documented *EGFR* mutations or *ALK* rearrangements were excluded to focus on patients who would be eligible for 1L IO therapies, which are generally indicated for patients with an *EGFR*- and *ALK*-negative status. Patients with other documented biomarkers, including *ROS1*, were included as 1L IO therapies were not counter-indicated for these patients at the time of the study.

The USON is a community-based network of 470 oncology clinics that treat over 1 million patients annually (The US Oncology Network 2018). Study data were primarily sourced from structured fields of the USON’s electronic healthcare record (EHR), iKnowMed (iKM), with supplemental vital status provided by the Social Security Administration’s Limited Death Access Master File. Patients who received care at USON clinics that did not use the full iKM capacities or who had data that were inaccessible for research purposes were excluded.

Patients with a diagnosis of NSCLC were identified through a review of iKM’s discrete diagnosis and histology fields, which were populated during the routine course of care. Patients with advanced disease status were identified by having ≥ 1 of the following indicators: (1) receipt of a numbered line of therapy; (2) stage IV disease; (3) indication of metastases based on tumor, node, metastases (TNM) stage; (4) location record of metastatic disease; or (5) current or prior disease status containing reference to advanced or metastatic disease.

The study was reviewed and granted an exception and waiver of consent by the US Oncology, Inc, Institutional Review Board. This study was conducted in accordance with the Declaration of Helsinki.

### Statistical analysis

Baseline characteristics at initiation of 1L treatment (overall and by treatment group) and treatment patterns, including distribution of regimens over time and sequences, were assessed descriptively. The regimens were classified as chemotherapy (eg, carboplatin+paclitaxel, carboplatin+pemetrexed), targeted therapy (bevacizumab+ carboplatin+paclitaxel, bevacizumab+carboplatin+pemetrexed), IO monotherapy (eg, pembrolizumab, nivolumab), or IO combination therapy (pembrolizumab+carboplatin+pemetrexed; Supplementary Table 1). Combination regimens were identified as treatments with overlapping treatment durations that were started within 14 days of each other. Time-to-event outcomes were assessed using the Kaplan-Meier method, with log-rank testing used to assess differences between the four 1L treatment groups.

Because it was not possible to assess dates of disease progression with the available data, time to treatment discontinuation (TTD) and time from 1L treatment initiation to 2L discontinuation or death were included; these measures were previously shown to be real-world proxies for progression-free survival (PFS) (Blumenthal et al. 2019; European Medicines Agency 2017). TTD was defined as the interval between 1L treatment initiation and discontinuation for any reason (including death).

OS was defined as the interval between 1L treatment initiation and the date of death (any cause). For time-to-event analyses, patients who did not experience the event during the study observation period were censored on the study end date or the last visit date available in the dataset, whichever occurred first.

As with any observational study, there was potential confounding when comparing treatment effects. Two methods were used to mitigate the bias. First, adjusted Cox regression analyses were performed to assess the relative effectiveness (ie, hazard ratio [HR]) of targeted therapy, IO monotherapy, and IO combination therapy vs chemotherapy, respectively, on OS and TTD, controlling for patient characteristics at baseline, including age, sex, body mass index (BMI), tobacco use, stage at diagnosis, ECOG PS, sites of metastasis, and histology. To construct these Cox models, baseline covariates and 1L treatment category were included in univariate models, and a stepwise selection approach was used to identify covariates for the multivariable models, with *P* ≤ .25 for entry and ≤ .15 for retention (Bursac et al. 2008).

Second, the inverse probability of treatment weighting (IPTW) method was applied to balance baseline demographic and clinical characteristics between the comparison cohorts (no limits were posed on these weights). To address the concern that patients treated under the different therapies have different characteristics, the propensity score method for multiple treatments was applied (McCaffrey et al. 2013). Specifically, generalized boosting models were used to generate propensity scores for each treatment group (available in the R package twang [Toolkit for Weighting and Analysis of Nonequivalent Groups]) (McCaffrey et al. 2013; RAND Corporation 2020).

Compared with a multinomial logistic model that is usually used to generate propensity scores for multiple treatment groups, the generalized boosted model is a nonparametric machine-learning classifying technique that has the advantage of automated variable selection and can provide more stable weights. In particular, multinomial logistic regression may require addition of many polynomial and cross product terms for covariates that are not balanced, and, in some cases, it may not be possible to test variation of possible polynomial and interaction terms. This approach also assumes appropriate candidate terms are tested in the model. Beyond the challenges of implementing this algorithm, linearity assumptions of logistic regression can lead to very small probabilities and extremely large weights (Harder et al. 2010; Kang and Schafer 2007; Lee et al. 2010).

The generalized boosted model was set up to achieve the best balance in covariates between each treatment group and the entire patient population. To obtain the optimum balance, the maximum number of regression trees was set to 10,000. In every iteration, the model with the additional tree was assessed to see if the balance measure was improved. The final model with the number of trees providing the best balance of baseline characteristics was selected. To assess the balance of each characteristic, the absolute standardized mean difference of effect size (standardized bias) or Kolmogorov-Smirnov statistic was estimated in tabular and graphical forms (Supplementary Figure 1 and Supplementary Table 2).

After IPTW was applied, Kaplan-Meier curves for OS and TTD were estimated, and a Cox model was used to estimate the HR of targeted therapy, IO monotherapy, and IO combination therapy vs chemotherapy.

As exploratory analyses, OS and TTD were estimated using Kaplan-Meier methods for the four 1L treatment categories and select baseline characteristics, including histology, ECOG PS, and tobacco use.

## Results

### Study population

**Fig. 1:**
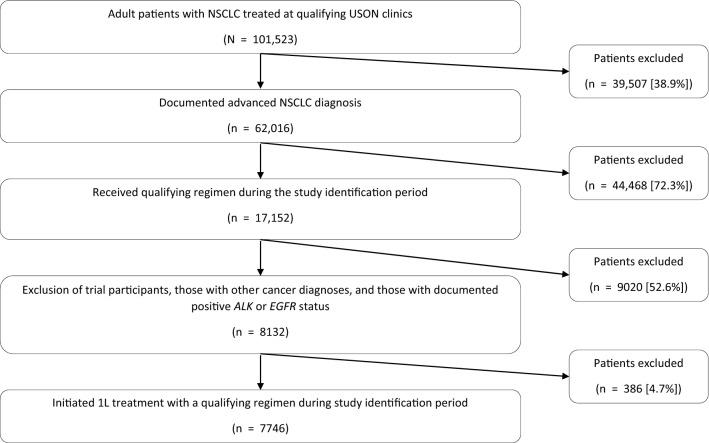
Study attrition

In total, 7746 patients met eligibility criteria and were included in the analysis, with a median follow-up of 7.2 months (range, 0.0-47.1 months; Fig. 1; Table 2). Across the study population, the median age was 68 years (range, 26–90+ years), with 55.0% male and 66.2% having nonsquamous histology. Most patients (64.2%) were diagnosed at stage IV. At initiation of 1L treatment, 64.1% had an ECOG PS of 0/1, 18.9% had a PS of 2+, and 17.0% lacked PS documentation. Most patients lacked documentation of biomarker mutational status.

**Table 2:** Baseline demographic and clinical characteristics

	Overall (*N* = 7746)	Systemic chemotherapies (*n* = 5859)	Targeted therapies (*n* = 656)	IO monotherapies (*n *= 907)	IO combination regimens (*n* = 324)
Follow-up, median (range), months	7.2 (0.0–47.1)	7.3 (0.0–47.1)	7.6 (0.0–47.1)	6.7 (0.0–46.1)	5.9 (0.0–42.4)
Time since initial NSCLC diagnosis to index, median (range), weeks	6.1 (0.0–1369.6)	6.0 (0.0–1369.6)	5.3 (0.0–907.6)	8.1 (0.0–860.3)	6.4 (0.3–856.3)
Time since advanced NSCLC diagnosis to index, median (range), weeks	2.9 (0.0–735.1)	2.4 (0.0–624.3)	3.1 (0.0–609.6)	4.0 (0.0–735.1)	4.1 (0.0–244.7)
Age at advanced NSCLC diagnosis, median (range), years	68 (26–90+)	68 (26–90+)	66 (29–89)	70 (27–90+)	68 (38–90+)
Male sex, *n* (%)	4261 (55.0)	3289 (56.1)	347 (52.9)	468 (51.6)	157 (48.5)
Race, *n* (%)					
Black/African American	768 (9.9)	599 (10.2)	62 (9.5)	83 (9.2)	24 (7.4)
White	6109 (78.9)	4640 (79.2)	517 (78.8)	712 (78.5)	240 (74.1)
Other	166 (2.1)	122 (2.1)	19 (2.9)	15 (1.7)	10 (3.1)
No information	703 (9.1)	498 (8.5)	58 (8.8)	97 (10.7)	50 (15.4)
Tobacco use, *n* (%)					
Never	801 (10.3)	536 (9.1)	123 (18.8)	95 (10.5)	47 (14.5)
Current	3400 (43.9)	2643 (45.1)	262 (39.9)	379 (41.8)	116 (35.8)
Former	3232 (41.7)	2439 (41.6)	240 (36.6)	404 (44.5)	149 (46.0)
No information	313 (4.0)	241 (4.1)	31 (4.7)	29 (3.2)	12 (3.7)
Body mass index, *n* (%)					
Underweight	479 (6.2)	353 (6.0)	34 (5.2)	73 (8.0)	19 (5.9)
Normal	3172 (41.0)	2371 (40.5)	272 (41.5)	390 (43.0)	139 (42.9)
Overweight	2349 (30.3)	1780 (30.4)	203 (30.9)	260 (28.7)	106 (32.7)
Obese	1700 (21.9)	1341 (22.9)	133 (20.3)	166 (18.3)	60 (18.5)
Not documented	46 (0.6)	14 (0.2)	14 (2.1)	18 (2.0)	0
Disease histology, *n* (%)					
Squamous cell carcinoma	2114 (27.3)	1834 (31.3)	12 (1.8)	242 (26.7)	26 (8.0)
Non-squamous cell carcinoma	5126 (66.2)	3629 (61.9)	588 (89.6)	629 (69.4)	280 (86.4)
Other	230 (3.0)	188 (3.2)	20 (3.0)	16 (1.8)	6 (1.9)
No information	276 (3.6)	208 (3.6)	36 (5.5)	20 (2.2)	12 (3.7)
Distant metastatic site(s), *n* (%)					
Brain	890 (11.5)	647 (11.0)	65 (9.9)	124 (13.7)	54 (16.7)
Bone	1472 (19.0)	1042 (17.8)	169 (25.8)	172 (19.0)	89 (27.5)
Liver	616 (8.0)	445 (7.6)	60 (9.1)	80 (8.8)	31 (9.6)
Lung	1205 (15.6)	849 (14.5)	123 (18.8)	160 (17.6)	73 (22.5)
Peritoneum	49 (0.6)	38 (0.6)	3 (0.5)	8 (0.9)	0
Other	3406 (44.0)	2402 (41.0)	359 (54.7)	467 (51.5)	178 (54.9)
None/no information	3178 (41.0)	2620 (44.7)	188 (28.7)	283 (31.2)	87 (26.9)
Stage at diagnosis, *n* (%)					
I–II	799 (10.3)	614 (10.5)	44 (6.7)	122 (13.5)	19 (5.9)
III	1652 (21.3)	1459 (24.9)	50 (7.6)	124 (13.7)	19 (5.9)
IV	4974 (64.2)	3543 (60.5)	531 (80.9)	629 (69.3)	271 (83.6)
No information	321 (4.1)	243 (4.1)	31 (4.7)	32 (3.5)	15 (4.6)
ECOG PS, *n* (%)					
0/1	4968 (64.1)	3774 (64.4)	420 (64.0)	565 (62.3)	209 (64.5)
2+	1462 (18.9)	1134 (19.4)	84 (12.8)	199 (21.9)	45 (13.9)
No information	1316 (17.0)	951 (16.2)	152 (23.2)	143 (15.8)	70 (21.6)
*EGFR* status, *n* (%)					
Negative	1271 (16.4)	721 (12.3)	114 (17.4)	342 (37.7)	94 (29.0)
Documented as unknown/no information	6475 (83.6)	5138 (87.7)	542 (82.6)	565 (62.3)	230 (71.0)
*ALK* status, *n* (%)					
Negative	1297 (16.7)	735 (12.5)	116 (17.7)	341 (37.6)	105 (32.4)
Documented as unknown/no information	6449 (83.3)	5124 (87.5)	540 (82.3)	566 (62.4)	219 (67.6)
*ROS1* status, *n* (%)					
Positive	39 (0.5)	2 (0.0)	36 (5.5)	0 (0.00)	1 (0.3)
Negative	1552 (20.0)	893 (15.2)	84 (12.8)	420 (46.3)	155 (47.8)
Documented as unknown/no information	6155 (79.5)	4964 (84.7)	536 (81.8)	487 (53.7)	168 (51.8)
PD-L1 status, *n* (%)					
Positive	504 (6.5)	57 (1.0)	13 (2.0)	16 (4.9)	418 (46.1)
Negative	425 (5.5)	307 (5.2)	21 (3.2)	68 (21.0)	29 (3.2)
Documented as unknown/no information	6817 (88.0)	5495 (93.8)	622 (94.8)	240 (74.1)	460 (50.7)

*ALK* anaplastic lymphoma kinase, *ECOG PS* Eastern Cooperative Oncology Group performance status, *EGFR* epidermal growth factor receptor, *IO* immuno-oncology, *NSCLC* non-small cell lung cancer, *PD-L1* programmed death-ligand 1, *ROS1* ROS proto-oncogene 1 receptor tyrosine kinase

### Treatment patterns

The highest proportion of patients received 1L systemic chemotherapy (*n* = 5859 [75.6%]), followed by IO monotherapies (*n* = 907 [11.7%)], targeted therapies (*n* = 656 [8.5%]), and IO combination regimens (*n* = 324 [4.2%]; Table 2, Supplementary Table 1, Supplementary Figure 2). During the study observation period, 3620 patients (46.7%) received 2L treatment (Supplementary Table 3). Of the 4126 patients who did not receive 2L treatment, 1871 (45.3%) died prior to 2L treatment, 266 (6.4%) had evidence of ongoing treatment, and 1989 (48.2%) were lost to follow-up for unknown reasons. On the basis of the structured data available for this analysis, it was not possible to determine if the patients lost to follow-up transitioned to care outside the USON, were admitted to hospice, or died but did not have a record of death.

Higher proportions of patients who received IO therapies (22.4% of IO monotherapy patients, 18.4% of IO combination therapy patients) had evidence of ongoing therapy at the end of the study observation period compared with those who received systemic chemotherapies (1.2%) or targeted therapies (7.4%; Supplementary Table 3). In total, 48.6%, 43.6%, 37.0%, and 34.8% of patients who received systemic chemotherapies, targeted therapies, IO monotherapies, and IO combination regimens, respectively, died before receiving 2L treatment.

**Fig. 2:**
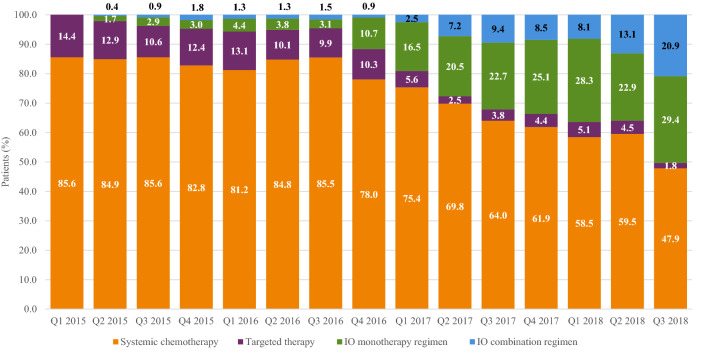
First-line treatment distribution over time

Figure 2 presents 1L treatment category distribution over time. In the first complete quarter of the study period (second quarter of 2015), 2.1% of 1L regimens included an IO therapy (1.7% monotherapy and 0.4% combination therapy). During the last complete quarter of the study period (the second quarter of 2018), 36.0% of 1L regimens contained an IO therapy (22.9% monotherapy and 13.1% combination therapy).

Across the 1L treatment groups, 48.2% (*n* = 2823) of patients who received systemic chemotherapies, 49.7% (*n* = 326) of patients who received targeted therapies, 78.4% (*n* = 710) of patients who received IO monotherapies, and 82.4% (*n* = 267) of patients who received IO combination regimens did not receive 2L treatment during the study observation period (Supplementary Figure 2). The most common treatment sequences were 1L systemic chemotherapy to 2L IO monotherapy (*n* = 2048) or 2L systemic chemotherapy (*n* = 636). Among patients who received 1L IO monotherapies, 15.7% (*n* = 142) received 2L systemic chemotherapy.

### Clinical outcomes

**Fig. 3:**
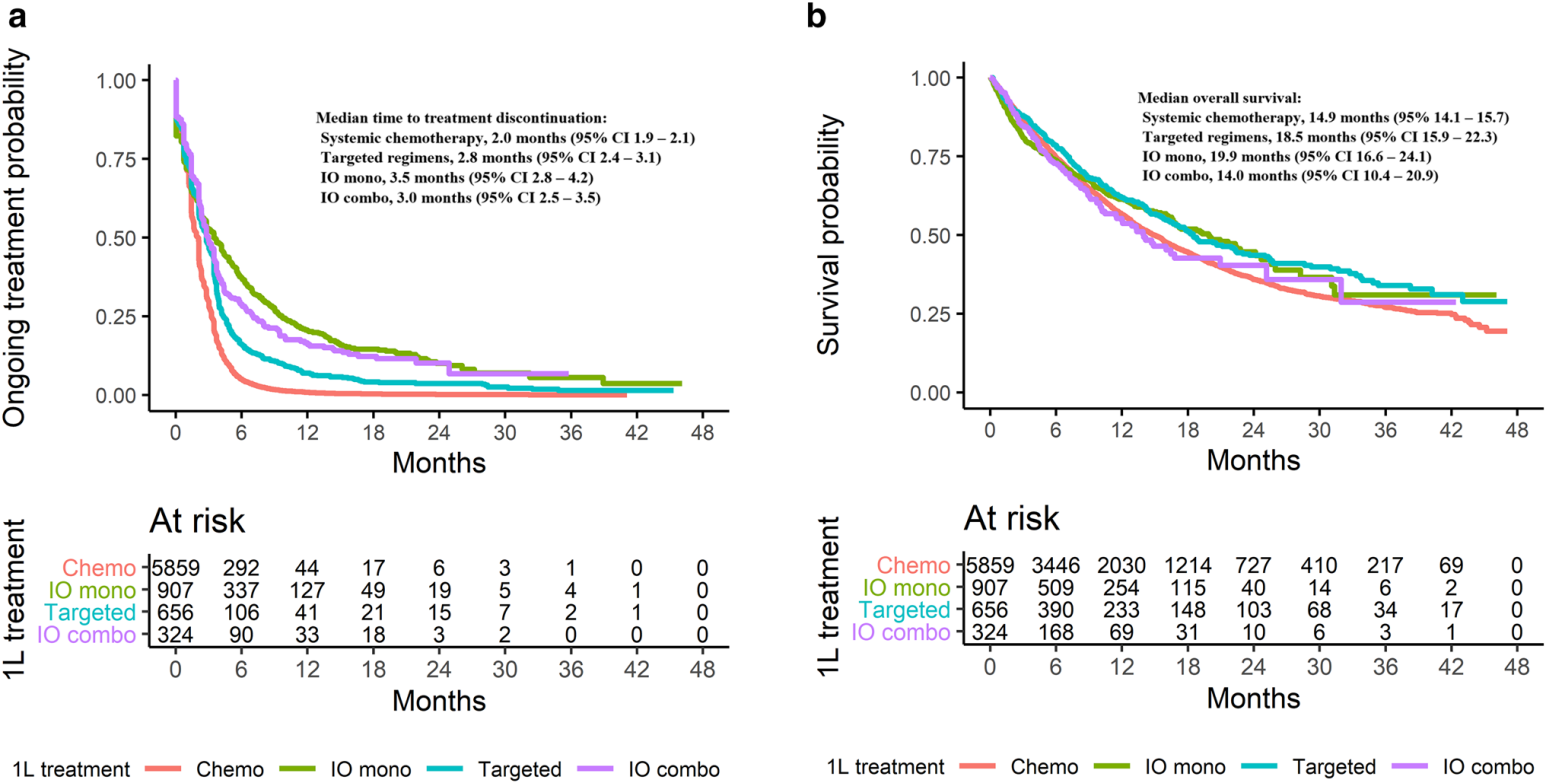
**a** Kaplan-Meier estimates of time to treatment discontinuation. **b** Kaplan-Meier estimates of overall survival

Median TTD was 2.0 months (95% CI, 1.9–2.1) with systemic chemotherapies vs 2.8 months (95% CI, 2.4–3.1) with targeted therapies (unadjusted HR, 0.633; 95% CI, 0.583–0.688), 3.5 months (95% CI, 2.8–4.2) with IO monotherapies (unadjusted HR, 0.405; 95% CI, 0.374–0.438), and 3.0 months (95% CI, 2.5–3.5) with IO combination regimens (unadjusted HR, 0.468; 95% CI, 0.414-0.529; Fig. 3a).

Median OS was 14.9 months (95% CI, 14.1–15.7) with systemic chemotherapies vs 18.5 months (95% CI, 15.9–22.3) with targeted therapies (unadjusted HR, 0.830; 95% CI, 0.733–0.941), 19.9 months (95% CI, 16.6–24.1) with IO monotherapies (unadjusted HR, 0.908; 95% CI, 0.810–1.019), and 14.0 months (95% CI, 10.4–20.9) with IO combination regimens (unadjusted HR, 1.041; 95% CI, 0.868–1.248; Fig. 3b). The survival rate at 42 months was 25.9% (95% CI, 23.9–27.9%) across the study population and 24.8% (95% CI, 22.7–27.0%) with systemic chemotherapies, 31.1% (95% CI, 24.6–37.9%) with targeted therapies, 31.0% (95% CI, 21.4–41.0%) with IO monotherapies, and 28.7% (95% CI, 14.6–44.6%) with IO combination therapies.

On the basis of multivariable Cox regression results, compared with patients who received 1L chemotherapy, lower risks of 1L discontinuation occurred among those who received targeted therapies (HR, 0.633; 95% CI, 0.581–0.689; *P* < .0001), IO monotherapy (HR, 0.390; 95% CI, 0.360–0.423; *P* < .0001), or IO combination therapy (HR, 0.466; 95% CI, 0.412–0.528; *P* < .0001; had lower risks of 1L discontinuation Table 3). Receipt of 1L targeted therapies and IO monotherapies was associated with a reduced risk of death compared with receipt of systemic chemotherapies (HR, 0.751; 95% CI, 0.660–0.855; *P* < .0001 and HR, 0.793; 95% CI, 0.706–0.890; *P* < .0001, respectively; Table 3).

**Table 3:** Treatment effect on time to treatment discontinuation and overall survival

Covariate	Level	Total	Event (censored)	Hazard ratio (95% confidence interval)		
				Unadjusted	Adjusted	IPTW adjusted
1L treatment	Systemic chemotherapy (reference)	5859	5822 (37)	–	–	–
	Targeted therapies	656	631 (25)	0.633 (0.583–0.688)	0.633 (0.581–0.689)	0.630 (0.607–0.654)
	IO monotherapy	907	747 (160)	0.405 (0.374–0.438)	0.390 (0.360–0.423)	0.410 (0.386–0.416)
	IO combination therapy	324	274 (50)	0.468 (0.414–0.529)	0.466 (0.412–0.528)	0.426 (0.410–0.442)
1L treatment	Systemic chemotherapy (reference)	5859	2796 (3063)	–	–	–
	Targeted therapies	656	270 (386)	0.830 (0.733–0.941)	0.751 (0.660–0.855)	0.688 (0.649–0.730)
	IO monotherapy	907	326 (581)	0.908 (0.810–1.019)	0.793 (0.706–0.890)	0.727 (0.688–0.768)
	IO combination therapy	324	122 (202)	1.041 (0.868–1.248)	0.933 (0.776–1.120)	0.937 (0.889–0.988)

Additionally, several baseline covariates were statistically associated with increased risk of 1L treatment discontinuation and/or increased risk of death (Table 4). Male sex, ECOG PS of 2+ or unknown (vs 0/1), documented metastases (lung, brain, bone, peritoneum, liver, or other), current or former tobacco use (vs never), and stage at diagnosis (stages IIA, IIB, IIIA, and no information vs IA) were associated with a significantly increased risk of treatment discontinuation. Obese BMI (vs normal), lung metastases, and squamous cell histology were associated with a significantly lower risk of discontinuation (all *P* < .05). Likewise, increased age, male sex, underweight BMI (vs normal), ECOG PS of 2+ or unknown (vs 0/1), documented metastases (brain, bone, liver, and other), and other histology (vs nonsquamous) were associated with a significantly increased risk of death, while obese and overweight BMI (vs normal) and lung metastases were associated with a significantly decreased risk of death (all *P* < .05).

**Table 4:** Multivariable Cox regression analysis of time to treatment discontinuation and overall survival

Time to treatment discontinuation					
Covariate	Level	Total	Event (censored)	Hazard ratio (95% confidence interval)	*P* value
1L treatment	Systemic chemotherapy (reference)	5859	5822 (37)	–	–
	Targeted therapies	656	631 (25)	0.633 (0.581–0.689)	< .0001
	IO monotherapy	907	747 (160)	0.390 (0.360–0.423)	< .0001
	IO combination therapy	324	274 (50)	0.466 (0.412–0.528)	< .0001
Sex	Female (reference)	3485	3344 (141)	–	–
	Male	4261	4130 (131)	1.053 (1.005–1.103)	.0302
BMI	Normal (reference)	3172	3064 (108)	–	–
	No information	63	60 (3)	1.204 (0.930–1.559)	.1583
	Obese	1686	1630 (56)	0.904 (0.851–0.961)	.0012
	Overweight	2343	2250 (93)	0.953 (0.902–1.007)	.0847
	Underweight	482	470 (12)	1.037 (0.940–1.143)	.4730
ECOG PS	0/1 (reference)	4968	4783 (185)	–	–
	2+	1462	1419 (43)	1.28 (1.206–1.359)	< .0001
	No information	1316	1272 (44)	1.097 (1.027–1.171)	.0057
Lung metastases	No (reference)	6541	6319 (222)	–	–
	Yes	1205	1155 (50)	0.910 (0.853–0.970)	.0041
Brain metastases	No (reference)	6856	6620 (236)	–	–
	Yes	890	854 (36)	1.092 (1.014–1.175)	.0203
Bone metastases	No (reference)	6274	6049 (225)	–	–
	Yes	1472	1425 (47)	1.125 (1.057–1.196)	.0002
Peritoneum metastases	No (reference)	7697	7425 (272)	–	–
	Yes	49	49 (0)	1.576 (1.188–2.091)	.0016
Liver metastases	No (reference)	7130	6875 (255)	–	–
	Yes	616	599 (17)	1.107 (1.016–1.208)	.0209
Other metastases	No (reference)	4340	4201 (139)	–	--
	Yes	3406	3273 (133)	1.105 (1.048–1.165)	.0002
Stage at diagnosis	IA (reference)	164	156 (8)	–	–
	No information	321	315 (6)	1.237 (1.005–1.522)	.0446
	IB	187	180 (7)	1.191 (0.961–1.477)	.1111
	IIA	235	226 (9)	1.316 (1.072–1.615)	.0086
	IIB	213	209 (4)	1.273 (1.034–1.568)	.0231
	IIIA	985	969 (16)	1.229 (1.036–1.458)	.0177
	IIIB	653	644 (9)	1.183 (0.992–1.411)	.0616
	IIIC	14	13 (1)	1.105 (0.627–1.947)	.7305
	IV	4974	4762 (212)	1.133 (0.965–1.331)	.1271
Tobacco use	Never (reference)	801	750 (51)	–	–
	Current	3400	3291 (109)	1.111 (1.024–1.205)	.0116
	Former	3232	3132 (100)	1.136 (1.047–1.232)	.0022
	Other	151	146 (5)	1.199 (1.003–1.434)	.0458
	No information	162	155 (7)	1.095 (0.919–1.305)	.3082
Disease histology	Non-squamous cell carcinoma (reference)	5126	4913 (213)	–	–
	Other	230	223 (7)	1.069 (0.934–1.224)	.3328
	Squamous cell carcinoma	2114	2066 (48)	0.938 (0.888–0.990)	.0202
	No information	276	272 (4)	0.965 (0.832–1.121)	.6433

Overall survival					
Covariate	Level	Total	Event (censored)	Hazard ratio (95% confidence interval)	Effect
1L treatment	Systemic chemotherapy (reference)	5859	2796 (3063)	–	–
	Targeted therapies	656	270 (386)	0.751 (0.660–0.855)	< .0001
	IO monotherapy	907	326 (581)	0.793 (0.706-0.890)	< .0001
	IO combination therapy	324	122 (202)	0.933 (0.776–1.120)	.4560
Age at advanced NSCLC diagnosis, years	Per year increase	7746	3514 (4232)	1.007 (1.003–1.011)	.0001
Sex	Female (reference)	3485	1499 (1986)	–	–
	Male	4261	2015 (2246)	1.183 (1.106–1.267)	< .0001
BMI	Normal (reference)	3172	1492 (1680)	–	–
	No information	63	20 (43)	0.992 (0.637–1.545)	.9713
	Obese	1686	708 (978)	0.808 (0.738-0.884)	< .0001
	Overweight	2343	1060 (1283)	0.895 (0.827–0.969)	.0059
	Underweight	482	234 (248)	1.246 (1.084–1.431)	.0019
ECOG PS	0/1 (reference)	4968	2202 (2766)	–	–
	2+	1462	790 (672)	1.689 (1.556–1.835)	< .0001
	No information	1316	522 (794)	1.182 (1.070–1.305)	.0010
Lung metastases	No (reference)	6541	2965 (3576)	–	–
	Yes	1205	549 (656)	0.873 (0.795–0.958)	.0044
Brain metastases	No (reference)	6856	3052 (3804)	–	–
	Yes	890	462 (428)	1.171 (1.056–1.298)	.0027
Bone metastases	No (reference)	6274	2698 (3576)	–	–
	Yes	1472	816 (656)	1.421 (1.306–1.546)	< .0001
Liver metastases	No (reference)	7130	3171 (3959)	–	–
	Yes	616	343 (273)	1.265 (1.126–1.421)	< .0001
Other metastases	No (reference)	4340	1818 (2522)	–	–
	Yes	3406	1696 (1710)	1.259 (1.165–1.360)	< .0001
Stage at diagnosis	IA (reference)	164	66 (98)	–	–
	No information	321	87 (234)	0.941 (0.668–1.326)	.7284
	IB	187	73 (114)	0.928 (0.665–1.296)	.6624
	IIA	235	94 (141)	0.880 (0.642–1.207)	.4281
	IIB	213	86 (127)	0.934 (0.677–1.289)	.6783
	IIIA	985	414 (571)	0.855 (0.658–1.111)	.2411
	IIIB	653	280 (373)	0.895 (0.683–1.172)	.4191
	IIIC	14	1 (13)	0.262 (0.036–1.891)	.1843
	IV	4974	2413 (2561)	1.209 (0.945–1.546)	.1321
Disease histology	Non-squamous cell carcinoma (reference)	5126	2347 (2779)	–	–
	Other	230	113 (117)	1.361 (1.126–1.646)	.0015
	Squamous cell carcinoma	2114	980 (1134)	1.001 (0.925–1.082)	.9869
	No information	276	74 (202)	0.943 (0.722–1.231)	.6652

*1L* first-line, *BMI* body mass index, *ECOG PS* Eastern Cooperative Oncology Group performance status, *IO* immuno-oncology

**Fig. 4:**
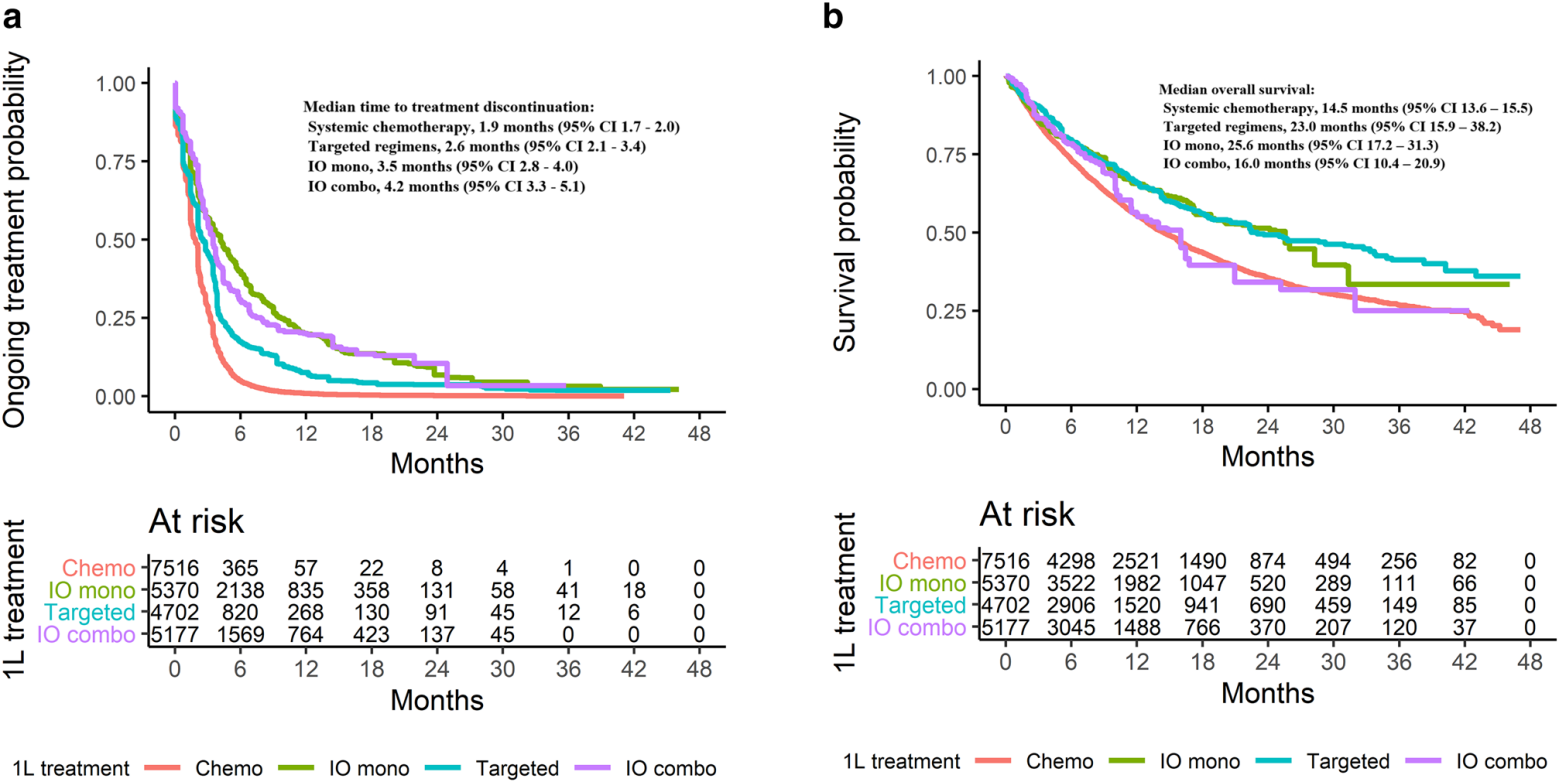
**a** Inverse probability weighting estimates of time to treatment discontinuation. **b** Inverse probability weighting estimates of overall survival

Figure 4a and b present the weighted Kaplan-Meier curves for TTD and OS by treatment groups. IPTW-adjusted median TTD was 1.9 months (95% CI, 1.7–2.0) with systemic chemotherapies vs 2.6 months (95% CI, 2.1–3.4) with targeted therapies (adjusted HR, 0.630; 95% CI, 0.607–0.654), 4.2 months (95% CI, 3.3–5.1) with IO monotherapies (adjusted HR, 0.410; 95% CI, 0.386–0.416), and 3.5 months (95% CI, 2.8–4.0) with IO combination regimens (adjusted HR, 0.426; 95% CI, 0.410–0.442; Fig. 4a). For TTD, the treatment groups diverged prior to 6 months after 1L initiation with IO monotherapies or IO combination regimens, demonstrating reduced risk of discontinuation compared with systemic chemotherapies and targeted therapies (Fig. 4a). IO monotherapy was associated with the longest TTD until 12 months when its Kaplan-Meier curve began overlapping with that of IO combination therapy.

Weighted median OS was 14.5 months (95% CI, 13.6–15.5) with systemic chemotherapies vs 23.0 months (95% CI, 15.9–38.2) with targeted therapies (adjusted HR, 0.688; 95% CI, 0.649–0.730), 25.6 months (95% CI, 17.2–31.3) with IO monotherapies (adjusted HR, 0.727; 95% CI, 0.688–0.768), and 16.0 months (95% CI, 10.4–20.9) with IO combination regimens (adjusted HR, 0.937; 95% CI, 0.889–0.988; Fig. 4b). OS across the groups was similar among targeted therapies and IO-based therapies for the first 10 months following 1L initiation, then diverged with targeted therapies and IO monotherapies demonstrating superior survival rates compared with systemic chemotherapy and IO combination regimens. Interestingly, after about 10 months, IO combination therapy showed similar OS to chemotherapy.

Kaplan-Meier estimates of OS and TTD by age, histology, ECOG PS, and tobacco use for each treatment group are presented in Supplementary Table 4. Overall, IO monotherapy showed superior TTD and OS compared with other therapies consistently across different subgroups. However, the improved TTD or OS tended to be smaller in the following subgroups: age ≥ 65 vs < 65 years, squamous cell carcinoma vs non-squamous cell carcinoma, ECOG PS 2+ vs 0/1, and never used tobacco vs current or former tobacco use.

## Discussion

To our knowledge, this is the latest study to investigate the real-world treatment patterns and clinical outcomes in patients with advanced NSCLC who initiated 1L treatment in a large network of community oncology practices. The demographic and clinical characteristics of this study population are similar to those reported in other real-world studies. A study by the Friends of Cancer Research (2018) compared patient characteristics and outcomes of patients with advanced NSCLC treated with IO therapies across 6 real-world databases (Friends of Cancer Research 2018). The median age at advanced NSCLC diagnosis across these databases ranged from 64 to 70 years. More than half (range, 50–56%) were male, with the majority being white (range, 65–87%). Also similar to our study, the Friends of Cancer Research (2018) found that most patients had a history of smoking (range, 78–92%), had been diagnosed with stage IV disease (range, 62–91%), and had non-squamous cell carcinoma (range, 66–74%). Khozin et al. (2018) observed similar characteristics in a multicenter analysis of data from The Flatiron Network: the median age was 69 years, 56% were male, and 64% were diagnosed with stage IV disease.

Biomarker status, especially that of PD-L1 expression, was not documented within the structured fields of the iKM EHR for the majority of the study population. In total, 5.6% of patients had a documented PD-L1–negative status, while 6.4% had a positive status. Khozin et al (2018) investigated IO use in patients with metastatic NSCLC and likewise observed that their PD-L1 status was rarely documented in the EHR. Among the 1344 patients included in their analysis, 8.0% had a record of PD-L1 testing. Future EHR-based studies should continue to monitor documentation of PD-L1 status because capture of this biomarker may increase over time as providers and payers adopt PD-L1 testing as standard practice for patients with NSCLC.

The treatment patterns observed in this study reflect the adoption of IO therapies in the US community oncology setting and are generally consistent with NCCN guideline recommendations. The first US Food and Drug Administration approval for 1L treatment of NSCLC with an IO agent was granted in October 2016 for pembrolizumab monotherapy in patients whose tumor cell PD-L1 expression was ≥ 50% (Pai-Scherf et al. 2017). With the release of additional clinical trial data, the use of IOs in the 1L setting has been expanded to include atezolizumab and pembrolizumab in combination with platinum-based chemotherapy regardless of the PD-L1 expression level (FDA approves atezolizumab with chemotherapy and bevacizumab for first-line treatment of metastatic non-squamous NSCLC (US Food and Drug Administration 2018a); FDA approves pembrolizumab in combination with chemotherapy for first-line treatment of metastatic squamous NSCLC (US Food and Drug Administration 2018b); FDA grants regular approval for pembrolizumab in combination with chemotherapy for first-line treatment of metastatic nonsquamous NSCLC (US Food and Drug Administration 2018c)).

Although previous real-world studies have shown high use of chemotherapy in the 1L setting, these studies were based on data preceding the advent of IO treatments. (Khozin et al. 2019a; Simeone et al. 2019) For example, in a retrospective analysis of patients diagnosed with stage IV or metastatic NSCLC from January 2013 to January 2017, Simeone et al (2019) found that platinum-based regimens accounted for most of the 1L therapies (61.1% of patients), while nivolumab comprised the most common 2L and 3L regimens (31.1% and 38.4% of patients, respectively). Similarly, in an analysis of patients treated with nivolumab or pembrolizumab from January 2011 through March 2016 for metastatic NSCLC in the Flatiron Health Network, the most common regimens preceding the earliest lines of IO treatment were platinum doublet–based therapies (62.1% of patients) (Khozin et al. 2019a).

In this study, the proportion of patients receiving 1L IO therapy increased over the study period, from 2.1% in the second quarter of 2015 to 36.0% in the second quarter of 2018. However, high proportions of patients continued to receive 1L systemic chemotherapies in 2018. Although IO combination therapies are being used more frequently, the initial approval for combination therapy was based on the KEYNOTE-189 trial results, which were released in the second quarter of 2018 (Gadgeel et al. 2020; Gandhi et al. 2018). Given the observation period of this study (1L initiation from March 1, 2015, to August 1, 2018), there was a limited period of time between the release of the clinical trial results and the study end date. Providers may have been cautious when adopting IO regimens into their practice; for example, they may have ordered IO therapies for their healthiest patients first because of the uncertainty about the clinical benefits and safety of these novel agents. Future research should consider whether there is increased adoption of IO therapies over a broader time period and, if not, what barriers might exist to more widespread use.

The apparently high attrition from 1L to 2L treatment in this study may reflect an unmet treatment need. Abernethy et al. 2017 reported a similar trend; of the patients who initiated 1L treatment in their study, 42% received 2L and 22% received 3L treatment during the observation period. Although reasons for treatment discontinuation were not assessed as part of this analysis, previous studies have shown that common reasons include disease progression, toxicity, and worsening PS (Gajra et al. 2018; Sztankay et al. 2017; Walker et al. 2017). Therefore, treatments that provide a sustained response with a favorable safety profile may allow patients to continue 1L treatment longer and eventually proceed to 2L treatment rather than discontinuing treatment altogether.

Differences in the proportions of patients in each treatment group who received 2L treatment were observed. Approximately 80% of patients who received IO regimens as 1L did not have evidence of advancement to 2L, compared with 50% of those who received chemotherapies or targeted therapies. Ongoing treatment at the end of the study period was one reason for non-advancement, along with death or becoming lost to follow-up (Supplementary Table 3). A higher proportion of patients who received IO therapy had ongoing treatment compared to those who received chemotherapies or targeted therapies, which is likely due to the approval history of IO regimens. Likewise, the proportion of patients who died prior to 2L treatment was lower in the IO treatment group than in the chemotherapy or targeted therapy groups.

The median TTD was significantly different across treatment groups (ranging from 2.0 months with systemic chemotherapies to 3.5 months with IO monotherapies; log-rank *P* value across all groups < .0001). IO-based regimens were associated with longer TTD than chemotherapies and targeted therapies, which might be due to better tolerability of IO therapies. Blumenthal et al (2019) reviewed 18 randomized clinical trials conducted after 2007 in patients with metastatic NSCLC. They found a TTD of 3.5 months with immune checkpoint inhibitors compared with 3.8 months with chemotherapy doublet monotherapy and 2.2 months with chemotherapy monotherapies. Similarly, Stewart et al. (2019) used 6 real-world databases to assess clinical outcomes of patients with advanced NSCLC treated with IO regimens (in any line of therapy) and found that the median TTD ranged from 3.21 to 7.03 months.

The median TTD across treatment groups in this study appeared to be comparable to that of prior studies of chemotherapies, targeted therapies, and IO-based regimens. However, as a real-world proxy of PFS, the estimates were markedly shorter than PFS estimates in clinical trials (Table 1). Additionally, the TTD in this study may be shorter than treatment durations observed in clinical trials. For example, Velcheti et al. (2019) compared the real-world time on treatment (rwTOT) vs TOT in KEYNOTE-024 and -042 among patients with metastatic NSCLC and an ECOG PS of 0/1 treated with 1L pembrolizumab. They reported a median rwTOT of 6.9 months vs a median TOT of 7.9 months in KEYNOTE-024 and 6.6 months in KEYNOTE-042. The median TTD of 3.5 months with IO monotherapy in our study was considerably shorter than the Velcheti et al (2019) estimates.

However, comparisons of these real-world results with clinical trials may reflect the underlying differences across patient populations and/or approaches to measurement. Specifically, more variation exists in the real-world settings with regard to patient and disease characteristics and clinical care practices, compared with the highly controlled care setting of clinical trials. For example, all patients had ECOG PS of 0/1 and stage IV disease in KEYNOTE-024, 042, and  407, as well as IMpower130 and  131, whereas our study population was not restricted to patients with favorable ECOG PS (Jotte et al. 2018, 2019; Mok et al. 2019a, b; Paz-Ares et al. 2018; Reck et al. 2016, 2019a, b; West et al. 2019). Also, PD-L1 status in this study was documented as unknown for nearly 90% of patients, while confirmation of PD-L1 status is frequently a selection criteria of IO clinical trials (Table 1).

The difference between the TTD in our study and the rwTOT in the Velcheti et al. (2019) study may reflect patient heterogeneity in our study. The median rwTOT among patients with ECOG PS of 2+ in the Velcheti et al. (2019) study was only 2.3 months, which is similar to the median TTD (2.1 months) observed for the same patient population in our study (Supplementary Table 4).

Few real-world studies have assessed OS in patients with advanced NSCLC who initiate 1L treatment in the era of IO therapies. Griffith et al. (2019) performed an EHR-based assessment of patients with advanced NSCLC who initiated 1L treatment between January 1, 2011, and February 28, 2018, and found a median OS of 10.98 months (95% CI 10.79-11.18) across the patient population. Similarly, Stewart et al. (2019) reported that median OS ranged from 8.67 (95% CI 6.83–10.02) to 15.78 months (95% CI 12.20–24.59) among patients with advanced NSCLC who received IO therapies across 6 real-world databases.

The median OS results in this study appear to be similar, although shorter, to some estimates reported in key clinical trials (Table 1). In particular, median OS among patients who received IO monotherapies was 19.9 months in our study, which was comparable to that of KEYNOTE-042 and IMpower110 (both IO monotherapy), and the median OS of 14.0 months associated with IO combination therapies in our study was similar to that of KEYNOTE-407, IMpower131, IMpower150, and CheckMate 227 (all IO combination therapy) (Hellmann et al. 2018, 2019; Jotte et al. 2018, 2019; Mok et al. 2019a, b; Paz-Ares et al. 2018; Socinski et al. 2018a, b; Spigel et al. 2019). However, comparisons of results for IO combination therapies must be made with caution, as the results for KEYNOTE-407 and IMpower131 were specifically for squamous disease and the results for IMpower150 were specifically for non-squamous disease (Jotte et al. 2018, 2019; Paz-Ares et al. 2018; Socinski et al. 2018a, b).

Differences in clinical outcomes by histology were observed in this study, with IO monotherapies having less impact on survival among patients with squamous cell carcinoma vs those with non-squamous cell carcinoma (Supplementary Table 4). KEYNOTE-407 reported that patients who received IO combination therapy (chemotherapy plus paclitaxel) exhibited a longer median OS than patients who received chemotherapy alone (HR, 0.64; 95% CI 0.49–0.85; *P* < .001), but mixed results were reported in IMpower131 (Jotte et al. 2018, 2019 Paz-Ares et al. 2018). IMpower131 contained 3 treatment arms: patients in arms A and B received atezolizumab and carboplatin with either paclitaxel or *nab*-paclitaxel, respectively, while those in arm C received carboplatin and *nab-*paclitaxel (Jotte et al. 2018, 2019) Patients in arm B demonstrated an improved PFS compared with those in arm C (HR, 0.715; 95% CI, 0.603–0.848; *P* = .0001), but this benefit was not observed in patients in arm A and was not replicated for OS.

Several factors were associated with a decreased risk of treatment discontinuation and death, including overweight BMI (vs normal). While the correlation between BMI and these treatment outcomes is unclear, it is possible that patients who experience minimal weight loss have less aggressive forms of disease or diminished levels of circulating cytokines associated with cachexia (An et al. 2020; Kichenadasse et al. 2019).

The results of the multivariable Cox regression analysis suggest a clinical benefit of IO therapies; in particular, IO monotherapy was significantly associated with a reduced risk of 1L treatment discontinuation and death (both *P* < .0001). This trend was also illustrated in the Kaplan-Meier curves adjusted by the IPTW method, which demonstrated that patients who received IO monotherapies had improved TTD and OS compared with those of systemic chemotherapies. However, the improved clinical outcomes associated with the IO monotherapies, especially OS, were most pronounced in specific patient subgroups (ie, patients with non-squamous cell carcinoma and those with ECOG PS of 0/1). Additional studies are needed to confirm the clinical benefit of IO monotherapy in other patient subgroups, such as patients with squamous cell carcinoma, older patients (aged ≥ 65 years), and those with former tobacco use (Supplementary Table 4).

While we did not specifically compare IO monotherapy to IO combination therapy, our results suggest that IO monotherapy was associated with more favorable survival rates than IO combination therapy, which exhibited survival trends similar to those of systemic chemotherapy, even after adjusting with IPTW. In KEYNOTE-024, median OS was 26.3 months (95% CI 18.3–40.4) among patients who received pembrolizumab monotherapy, compared to 22.0 months (95% CI 19.5, 25.2) among patients who received pembrolizumab combination therapy in KEYNOTE-189 trial, (Table 1); (Gadgeel et al. 2020; Reck et al. 2016, 2019a, b). Because IO combination therapies are being more frequently used after recent approval, future studies should determine if uncontrolled patient- or treatment-related factors, such as PD-L1 status, toxicity, management of toxicity, as well as sample size, may have influenced these outcomes.

This study is subject to several limitations. First, the low capture of PD-L1 information limited the assessment of outcomes by PD-L1 status, and the results may have been confounded by inclusion of *EGFR*- or *ALK*-positive patients who had a missing/unknown status in their EHR. An exploratory analysis was performed to assess exclusion of the small proportion of patients who received targeted therapies indicated for 1L treatment of patients with *EGFR* mutations or *ALK* rearrangements (*n* = 116 [1.5% of the study population]). The results of this analysis did not yield meaningful differences in the baseline characteristics, TTD, or OS of the study population (data not shown); thus, these patients were retained for the final analyses, as their biomarker status could not be confirmed.

Additionally, because this was a retrospective observational study, patients were not assigned to treatment groups, and underlying clinical differences may have influenced outcomes. The sample size was also unbalanced across the treatment groups, with a substantially higher proportion of patients receiving 1L systemic chemotherapy than the other 1L treatments. To reduce bias due to patient heterogeneity, adjusted analyses were performed to control for selected covariates, although unobserved factors (eg, PD-L1 status) may have confounded the results.

Approximately 50% of patients did not receive 2L treatment for unknown reasons. These patients may have received 2L treatment outside the USON, or their death date was not captured; as a result, TTD and OS for these patients may be misrepresented. While the proportions of patients who did not receive 2L treatment for unknown reasons were similar, it is unclear how patients without follow-up information may have influenced the study results.

Finally, differences in USON treatment practices or the patient population may have influenced the results. Specifically, the USON encourages the use of evidence-based treatment that reflects a refinement of the NCCN guidelines (Hoverman et al. 2014; National Comprehensive Cancer Network McKesson 2019). Although USON clinics are located across the US, a high proportion of clinics are located in southern regions. As such, USON patients may be different than other community oncology patient populations or patients treated in academic settings. Thus, the results of this study are most generalizable to community-based oncology clinics that also adhere to best-practice guidelines consistent with NCCN guidelines.

By sourcing data from a large network of community-based oncology clinics, this study provides insights into patient profiles, treatment patterns, and outcomes in a large population of patients with advanced NSCLC. These results supplement findings from clinical trials, as patients who were not enrolled in clinical trials were included in the analysis. Therefore, treatment trends and clinical outcomes reflect how current therapeutic options are being used in the community oncology setting.

## Conclusions

These results provide insight into current treatment use and outcomes associated with 1L treatment of advanced NSCLC in a real-world setting. Although the treatment landscape appears to be shifting in response to expanded approvals for IO therapies, a high proportion of patients still receive systemic chemotherapy, and many do not appear to advance to 2L treatment. Our results indicate that targeted therapies and IO monotherapies were associated with the most favorable clinical outcomes; the longest median TTD and OS occurred with IO monotherapies. However, the clinical benefit of IO monotherapies may be limited in certain patient subgroups, including those with squamous cell carcinoma and ECOG PS of 2+. Unlike IO monotherapy, IO combination therapy appeared to be associated with similar survival trends as systemic chemotherapy. This finding may have been influenced by underlying differences between the treatment subgroups that could not statistically be controlled (eg, PD-L1 expression).

The results of this study suggest that patients treated in this real-world setting exhibited similar, but shorter, median OS and markedly lower median TTD compared with PFS in clinical trials. The lower TTD observed in this study may be due to differences in measurement, effectiveness, and/or tolerability of treatment in a heterogeneous population of patients treated in the community oncology setting. Overall, these findings suggest the continued need to develop safe and effective treatments for advanced NSCLC. As these therapeutic advancements are released, future studies should continue to use available real-world data to monitor outcomes associated with 1L regimen choice.

## Electronic supplementary material

Below is the link to the electronic supplementary material.

Supplementary file1 (DOCX 106 kb)

Supplementary file2 (DOCX 143 kb)

## Data Availability

The health data used to support the findings of this study are restricted by the US Oncology Institutional Review Board in order to protect patient privacy. For this reason, data used to support the findings of this study have not been made available.

## Code availability (software application or custom code)

Statistical coding used to conduct the analyses is proprietary and, as such, has not been made available.
