# Supplementary material for: Treatment patterns and clinical outcomes in patients with advanced non-small cell lung cancer initiating first-line treatment in the US community oncology setting: a real-world retrospective observational study
Source: J Cancer Res Clin Oncol. 2020 Dec 2;147(3):671–90. doi: 10.1007/s00432-020-03414-4 (PMC7873014; doi:10.1007/s00432-020-03414-4)
Supplement: Supplementary file 1 — Supplementary file1 (DOCX 106 kb) [file 432_2020_3414_MOESM1_ESM.docx]

**Supplementary Figure 1. Absolute Standard Difference of Patient Characteristics Before and After Propensity Weighting**

**Abbreviations:** es.mean, effect size mean; ks.mean, Kolmogorov–Smirnov mean.
