# Supplementary material for: Treatment patterns and clinical outcomes in patients with advanced non-small cell lung cancer initiating first-line treatment in the US community oncology setting: a real-world retrospective observational study
Source: J Cancer Res Clin Oncol. 2020 Dec 2;147(3):671–90. doi: 10.1007/s00432-020-03414-4 (PMC7873014; doi:10.1007/s00432-020-03414-4)
Supplement: Supplementary file 2 — Supplementary file2 (DOCX 143 kb) [file 432_2020_3414_MOESM2_ESM.docx]

**Supplementary Figure 2. First- Through Third-Line Treatment Sequences**


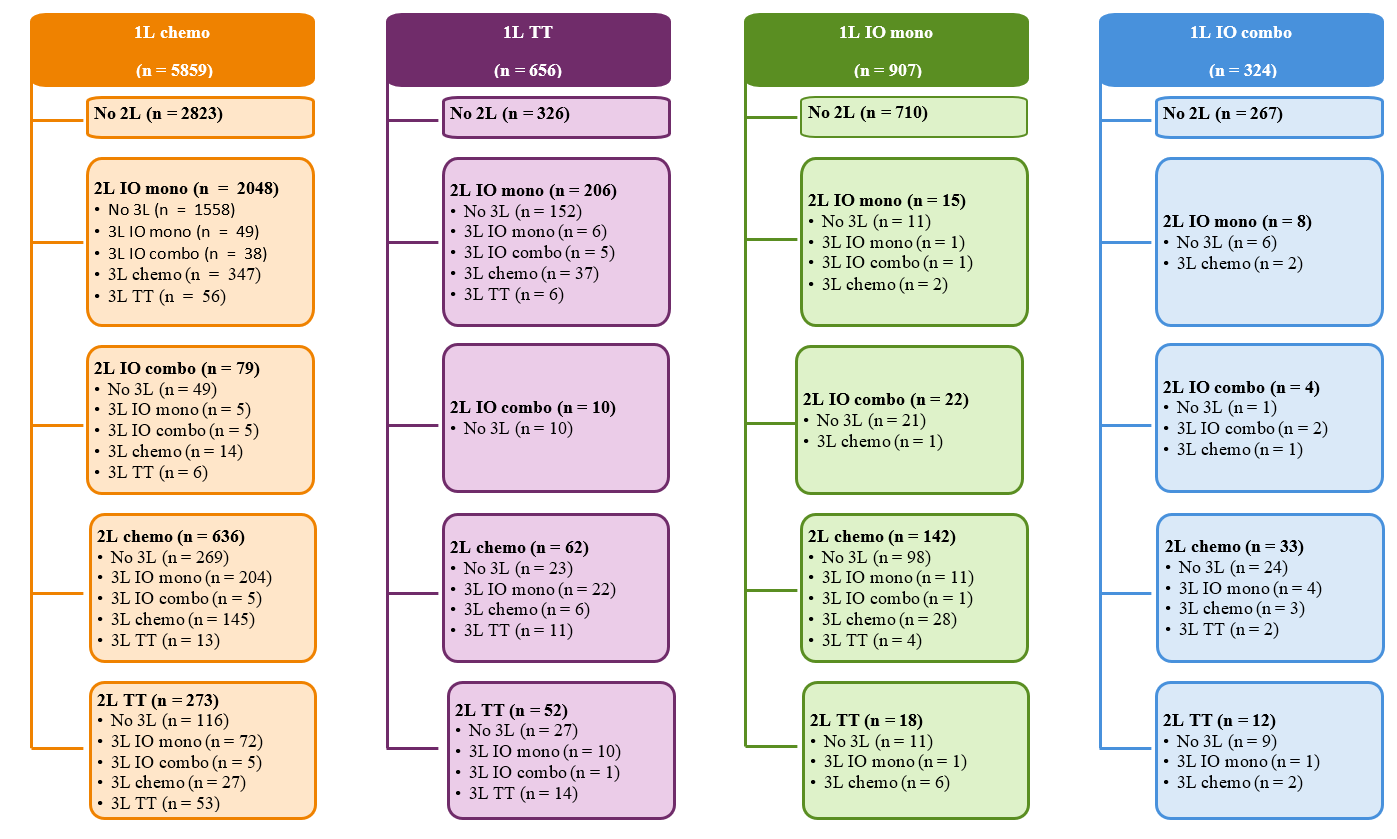


**Abbreviations:** 1L, first-line; 2L, second-line; 3L, third-line; chemo, systemic chemotherapy; IO combo, immuno-oncology combination regimens; IO mono, immuno-oncology monotherapies; TT, targeted therapy.
